# Effects of drought stress during critical periods on the photosynthetic characteristics and production performance of Naked oat (*Avena nuda* L.)

**DOI:** 10.1038/s41598-022-15322-3

**Published:** 2022-07-01

**Authors:** Xinjun Zhang, Wenting Liu, Yaci Lv, Tianliang Li, Jianzhao Tang, Xiaohong Yang, Jing Bai, Xin Jin, Haitao Zhou

**Affiliations:** 1 Zhangjiakou Academy of Agricultural Sciences, Zhangjiakou, 075000 China; 2 Hengshui University, Hengshui, 053000 China; 3 Engineering Technology Research Center, Geographic Information Development and Application of Hebei, Institute of Geographical Sciences, Hebei Academy of Sciences, Shijiazhuang, 050011 China; 4 Beijing Normal University, Beijing, 100875 China

**Keywords:** Plant sciences, Photosynthesis, Plant physiology, Plant stress responses

## Abstract

Revealing the effects of drought stress on the photosynthetic characteristics and yield of naked oats (*Avena nuda* L.) is significant for enhancing the productivity of oats. In this study, a potted experiment consisting of four water levels was conducted in the Bashang area of Hebei Province, China. The drought stress period was established as the continual 8 days during the jointing-heading stage. The aims were to reveal the impacts of drought stress on the photosynthetic characteristics and yield of naked oats during the critical stage. The results showed that the photosynthetic rate (*P*_n_), transpiration rate (*T*_r_), and stomatal conductance (*G*_s_) decreased under all conditions of drought stress. The intercellular CO_2_ concentration (*C*_i_) decreased under light drought stress, while it increased under moderate and severe drought stress. The initial chlorophyll fluorescence rate (*F*_o_) increased by 9.03–50.92% under drought stress, and the maximum fluorescence rate (*F*_m_) decreased by 8.49–19.73% under drought stress. The photochemical efficiency (*F*_v_/*F*_m_) increased by 10.37–24.12% under drought stress. The yields decreased by 9.5–12.7%, 16.8–27.0% and 44.1–47.7% under light, moderate and severe drought stress during the critical stage, respectively. The grains per panicle decreased by 1.7–12.5%, 8.3–24.3% and 32.7–34.2% under light, moderate and severe drought stress conditions, respectively. The 1000-grain weight decreased by 5.7–8.6%, 12.7–14.5% and 16.8–19.1% under light, moderate and severe drought stress conditions, respectively. The panicle numbers did not vary significantly among the different drought stress treatments. The photosynthetic rate, stomatal conductance and transpiration all had significant positive relationships with the yield of naked oat (*P* < 0.01). Parameters of PS II except for *F*_o_ all had significant positive relationships with the yield of naked oats (*P* < 0.05). This study is significant for enhancing the production efficiency of naked oat under drought stress.

## Introduction

Naked oat (*Avena nuda* L) is a traditional food, feed and forage crop in north China^1^. Owing to its rich content of nutrients, it is significant for maintaining human health^2^. However, oat crops use large amounts of water, and they require more water during their growth period than other cereal crops^3^. Drought stress significantly impacts oat production, particularly during critical growing periods^4^. Previous studies showed that water shortages decrease the photosynthetic rate, stomatal conductance and intercellular CO_2_ content^5,6^. Moreover, research also showed that water stress at each stage could reduce the grain weight per plant, number of effective ears, number of spikelet and 1000 grains weight, which resulted in the shrinkage of grain, and a decrease in yield^7^.

Photosynthesis is the basis for dry matter accumulation and yield formation^8^. However, drought stress damages the physiological metabolism and photosynthesis of plants^9–11^, which reduces crop production^12–14^. Leaves are the primary photosynthetic organ, and their photosynthetic capacity is primarily influenced by water conditions^15,16^. Photosystem II (PS II) is the most sensitive to leaf damage^16^. Under water stress, the light use efficiency and photosynthetic capacity of oat leaves decrease, which results in a decrease of the overall performance of PS II, and results in a decrease in dry matter accumulation^17,18^.

The Bashang area of Hebei Province is one of the staple areas for the production of naked oats. However, water shortages, characterized by low and highly variable amounts of precipitation, are the major limiting factor for oat production in this region^19,20^. Some studies were conducted to evaluate the impacts of drought stress on oat production in this region. Ge^21^ studied the photosynthetic performance under different water stress conditions in the Bashang area and found that with the extension of drought stress time and the aggravation of degree of drought stress, the primary limiting factors for the photosynthetic rate changed from stomatal factors to non-stomatal factors.

However, evaluations based on the structure and functions of light system have rarely been conducted in this region. Therefore, the objectives of this study were the following: 1) to reveal the responses of photosynthetic characteristics, including the net photosynthetic rate (*P*_n_), stomatal conductance (*G*_s_), intercellular CO_2_ concentration (*C*_i_), transpiration rate (*T*_r_) and chlorophyll fluorescence characteristics of naked oat under different levels of drought stress in a critical period; and 2) to understand the responses of naked oat yield, biomass and yield components to different levels of drought stress during a critical period.

## Materials and methods

### Study site, climate and soil data

**Figure 1:**
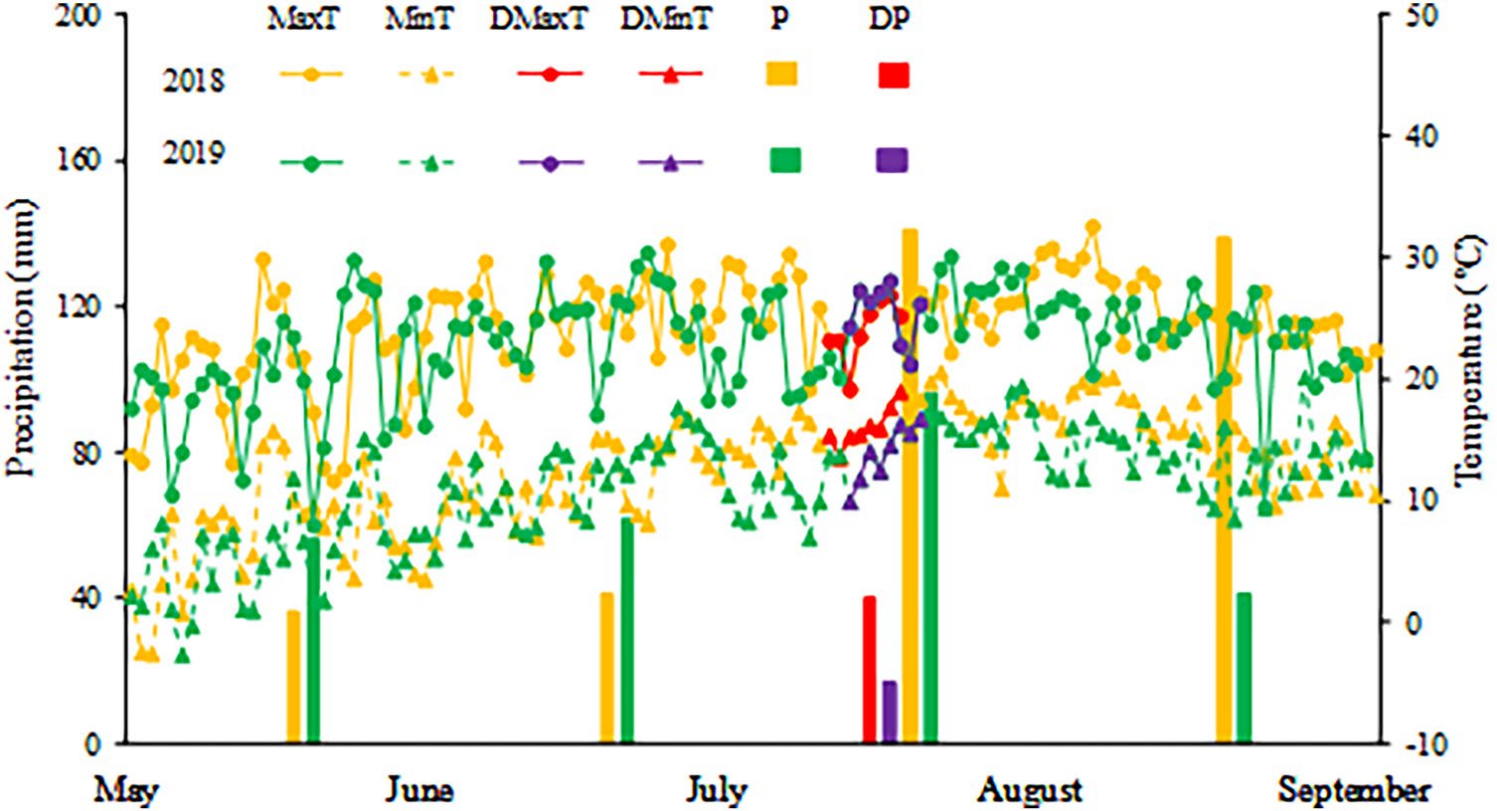
The maximum temperature (MaxT), minimum temperature (MinT) and month total precipitation (P) during the naked oat growing season, and maximum temperature (DMaxT), minimum temperature (DMinT) and total precipitation (DP) during the drought stress periods in 2018 and 2019 in the Bashang area, Hebei Province, China.

The experiment was conducted at the Xishungou Station (41° 3′ 54″ N, 114° 4′ 18″ E) of the Zhangjiakou Academy of Agricultural Sciences (Zhangjiakou, China) from 2018 to 2019. The station is characterized by a typical continental climate with abundant solar radiation, warm summers and cold winters. The soil type is chestnut soil, and the detailed soil information is shown in Table 1. The average daily maximum and minimum temperatures were 24.2 °C and 12.8 °C in 2018, respectively, while these values were 23.0 °C and 11.2 °C in 2019, respectively (Fig. 1). Total precipitation during the growing season was 387.6 mm and 238.3 mm in 2018 and 2019, respectively (Fig. 1). The daily maximum temperature during the drought stress treatment period was lower in 2018 with a range of 19.2–26.9 °C, and the range was 21.2–28.1 °C in 2019 (Fig. 1). However, the daily minimum temperature was higher in 2018, with a range of 13.6–19.0 °C, and the range was 10.0–16.8 °C in 2019 (Fig. 1). Moreover, the variation of temperature during the treatment period was higher in 2019 (Fig. 1). The precipitation during the period was higher in 2018 with a value of 40.1 mm, and this value was 12.2 mm in 2019 (Fig. 1).

**Table 1:** Vertical distribution of the physical and chemical properties of soil in the study site.

Year	Soil depth (cm)	Available N (mg/kg)	Available P (mg/kg)	Available K (mg/kg)	Organic carbon (g/kg)	pH value
2018	0–20	50.92	29.97	55.21	10.23	6.96
	20–40	53.97	19.39	42.40	9.24	6.95
2019	0–20	48.68	20.66	39.38	10.15	7.12
	20–40	52.75	17.93	46.92	12.00	7.10

### Experimental design

**Figure 2:**
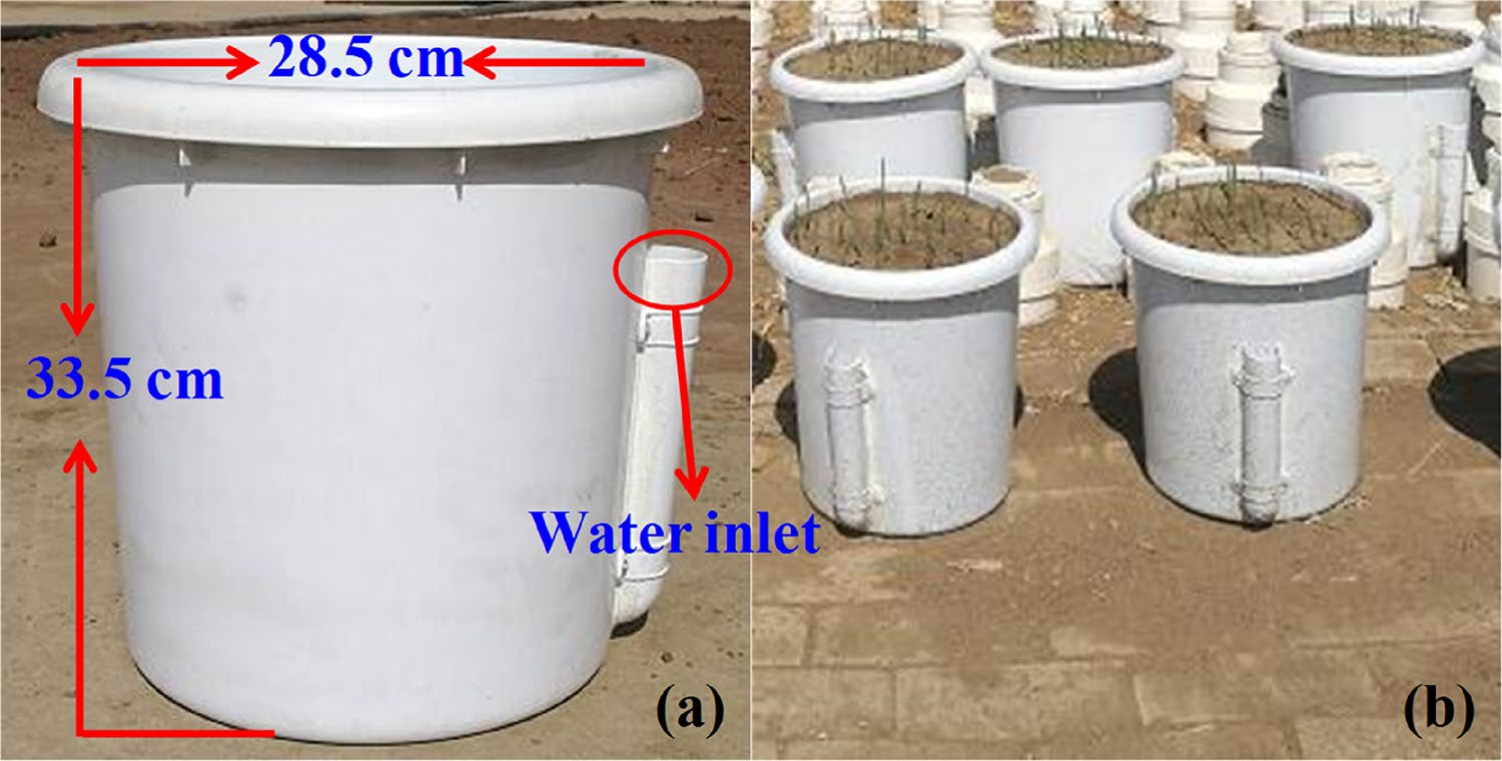
The plastic pots used in the experiment (**a**) and the pots planted with naked oat (**b**).

To explore the impact of drought stress on the photosynthetic characteristics and production performance of naked oat, potted experiments with four water levels were conducted in 2018 and 2019. To better understand the impact of drought stress on naked oat production and provide suggestions for oat production in the region, the cultivar Huazao2 was planted, which was bred by the Zhangjiakou Academy of Agricultural Sciences and approved by the Crop Variety Examination and Approval Committee of Hebei Province in 2001, with approval number: 200003. It is a cultivated genotype, and the collection of plant material complied with the relevant institutional, national, and international guidelines and legislation. This cultivar is resistant to drought stress, and it has narrow leaves, which ensure less water loss, and well-developed roots, which more easily absorb water from the soil. The results of regional trials showed that it is suitable for planting in dry land in north and northwest China. In addition, the cultivar is the most popular one used in the Bashang area. The seeds were sown on May 30 and June 2 in 2018 and 2019, respectively. Plastic pots with the same specifications (inner diameter 28.5 cm and 33.5 cm deep) were used to plant the materials, which weighed 0.85 kg, and each pot was filled with 23.15 kg of air-dried chestnut soil (Fig. 2). A plastic pipe was connected to the bottom of pot, and the top of the pipe was established as a water inlet (Fig. 2). The soil moisture could then be controlled by injecting water from the inlet (Fig. 2). The maximum soil water holding capacity was tested by the cutting ring method, and the soil nutrient parameters described in Table 1 were tested by the Hebei Academy of Sciences (Shijiazhuang, China).

Drought stress has significant impacts on the production of oats, particularly during critical growing periods, such as 12–15 days before heading ^1^. Therefore, the drought stress period was established as a continual 8-day period during the jointing-heading stage. Four treatments were established in the experiment: normal water supply (CK), light drought stress (LS), moderate drought stress (MS) and severe drought stress (SS), and the relative water content of the soil was 70–80%, 60–70%, 50–60% and 35–45% of the maximum soil water capacity, respectively. Six repetitions, i.e., six pots were established in each treatment, and 20 seedlings per pot were planted. The water control was initiated at approximately 20 days before the heading stage. The water was replenished at 8:00 and 18:00 every day, controlled by weighing and recorded. This lasted for 7 days after the expected level of drought stress was reached. The photosynthetic rate and chlorophyll fluorescence were measured for three consecutive days. After that, all the treatments were restored to the normal watering level. Except for the obvious difference of soil moisture in the pot, the other management treatments were kept the same. Three staple plants were selected for measurement and marked with plastic tags in each repetition, and the indices were measured with the third leaf counted from the tip of plant. There were 18 biological repetitions per treatment. The economic and yield characters of the naked oats were measured after the plants had matured and been harvested.

### Measurements

#### Photosynthetic rate

The photosynthetic rate of the naked oats leaves was measured at 09:00–11:00 on sunny days using an LC pro^+^ portable full-automatic photosynthetic measurement system (BioScientific, Ltd., Hertfordshire, UK) under natural conditions. The third healthy functional leaf counted from the tip of each staple plant was selected for measurements, and three data points within the same leaf were recorded for each measurement. The parameters obtained were the net photosynthetic rate (*P*_n_), stomatal conductance (*G*_s_), intercellular CO_2_ concentration (*C*_i_) and transpiration rate (*T*_r_).

#### Chlorophyll fluorescence

The chlorophyll fluorescence value of naked oats was determined using a Fluorpen FP100 chlorophyll fluorescence meter (Drásov, Czech Republic). The third healthy functional leaf counted from the tip of each staple plant was selected, and each three data points were measured within the same leaf. The parameters obtained were the initial fluorescence value (*F*_o_), the maximum fluorescence value (*F*_m_), and the light energy conversion efficiency *F*_v_/*F*_m_ of the PS II center.

#### The yield and yield components of oat

Ten plants that had been harvest in the mature stage were sampled from each pot, and the plant height, ear length, spikelet number, ear grain weight, stem number, ear number, ear grain number, and 1000 grain weight were measured. After the seed test, the grain yield and biomass were measured, and the remaining 10 plants in the pot were combined to calculate the total grain yield and biomass of 20 plants in each pot.

### Statistical analysis

#### The significance test for the values measured

The difference among the measured values under different treatments was tested based on a Duncan’s test.

#### Calculation of tolerance index and mean productivity

To evaluate the impacts of drought stress on naked oat yield, the Tolerance index (TOL)^22^ and Mean Productivity (MP)^23^ were utilized, and the equations were calculated as follows:

1 $$ {\text{TOL}} = Y_{{\text{c}}} - Y_{{\text{s}}} $$

2 $$ {\text{MP}} = \frac{{Y_{{\text{c}}} + Y_{{\text{s}}} }}{2} $$

where *Y*_c_ and *Y*_s_ are the yields under controlled and water stress conditions.

#### Calculations of the correlations between photosynthetic characteristics and naked oat yield

The correlations between photosynthetic characteristics and naked oat yield were calculated based on Pearson's correlation coefficients:

3 $$ R = \frac{{{\text{Cov}}\left( {X_{{\text{i}}} ,{ }Y} \right)}}{{{\upsigma }_{{X{\text{i}}}} \times {\upsigma }_{Y} }} $$

where *X*_i_ and *Y* are the photosynthetic indices and naked oat yield. Cov(*X*, *Y*) is the covariance between the photosynthetic indices and naked oat yield, and *σ*_Xi_ and *σ*_Y_ are the standard deviations of photosynthetic indices and naked oat yield.

#### Tools for data analysis and plotting

SPSS 10.0 (SPSS, Inc., Chicago, IL, USA) was used for data analysis, and Microsoft Office Excel 2010 (Redmond, WA, USA) was used for plotting.

## Results

### Effects of drought stress on photosynthetic characteristics

**Figure 3:**
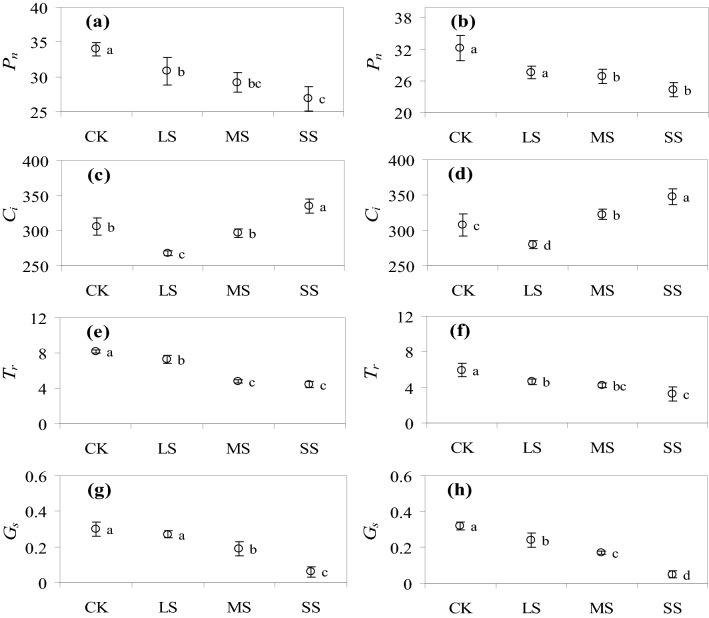
The photosynthetic rate (*P*_n_, μmol m^−2^ s^−1^), intercellular CO_2_ concentration (*C*_i_, μmol mol^−2^), transpiration rate (*T*_r_, mmol m^−2^ s^−1^) and stomatal conductance (*G*_s_, mol m^−2^ s^−1^) under the control treatment (CK), low drought stress (LS), moderate drought stress (MS) and severe drought stress (SS) in 2018 and 2019. (**a**, **c**, **e** and **g**) represent the values in 2018, while (**b**, **d**, **f** and **h**) represent the values in 2019. Different letters above the columns indicate significant differences (*P* < 0.05) determined by a Duncan’s test between treatments. The same letters above the error bars indicate no significant different at *P* > 0.05.

The photosynthetic rates under LS, MS and SS were significantly lower than those of the CK (*P* < *0.05*), and the values decreased by 9.30%, 14.08% and 20.89%, respectively, in 2018, while the decreases in 2019 were 14.43%, 16.85% and 24.55%, respectively (Fig. 3a,b). The photosynthetic rate was lower in 2019 than in 2018 for each treatment (Fig. 3a,b). With the increase in drought stress, the *C*_i_ first decreased and then increased (Fig. 3c,d). Compared with the CK, the *C*_i_ decreased by 12.41% under LS, while it increased by 9.49% under SS, the difference was not significant under MS in 2018 (Fig. 3c). In 2019, the *C*_i_ under LS increased significantly. The *C*_i_ under MS and SS was 4.94% and 13.09% higher than that of the CK (*P* < 0.05), respectively (Fig. 3d). The *C*_i_ in each treatment was higher in 2019 than in 2018 (Fig. 3c,d). With the increase in drought stress, the *T*_r_ gradually decreased (Fig. 3e,f). The *T*_r_ under LS, MS and SS during the 2-year period was significantly lower than that of the CK (*P* < 0.05). The *T*_r_ decreased by 10.83%, 41.39% and 45.61% in 2018, respectively (Fig. 3e), and by 21.85%, 29.30% and 45.77% in 2019, respectively (Fig. 3f). The *T*_r_ was lower in 2019 under all the treatments than in 2018 (Fig. 3e,f). The *G*_s_ of naked oats decreased as the intensity of drought stress increased (Fig. 3g,h). In 2018, the difference of *G*_s_ under LS and CK was not significant, while the *G*_s_ decreased by 35.68% and 78.89% in MS and SS, respectively, compared with that of the CK (Fig. 3g). In 2019, the Gs under LS, MS and SS were 26.04%, 46.88% and 85.42% lower than that of the CK, respectively (*P* < 0.05) (Fig. 3h). The *G*_s_ was higher under the CK in 2019 than that in 2018, while the values in other treatments were higher in 2018 (Fig. 3g,h).

### Effects of drought stress on photosystem II

**Figure 4:**
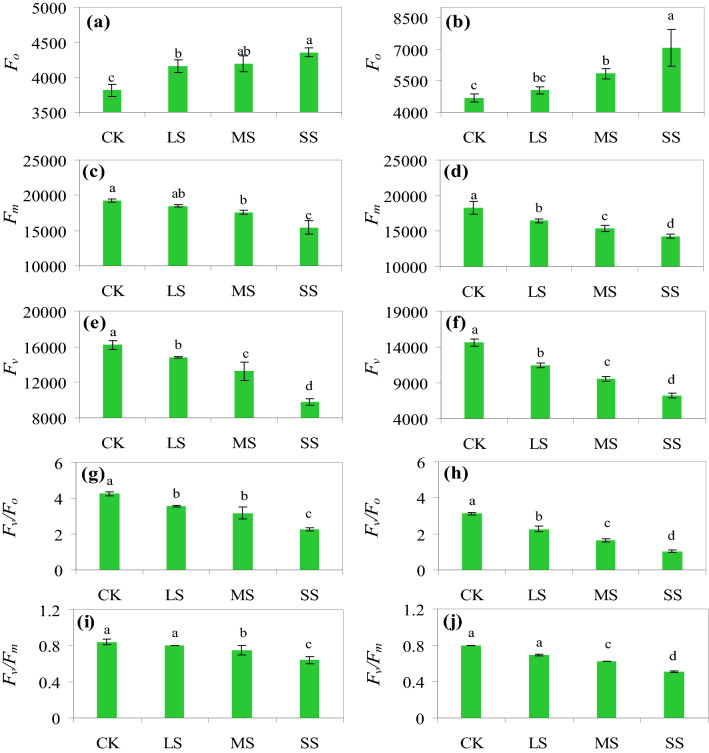
The initial fluorescence value (*F*_o_), maximum fluorescence value (*F*_m_), *F*_v_/*F*_o_ and *F*_v_/*F*_m_ under the control treatment (CK), low drought stress (LS), moderate drought stress (MS) and severe drought stress (SS) in 2018 and 2019. (**a**, **c**, **e** and **g**) represent the values in 2018, while (**b**, **d**, **f** and **h**) represent the values in 2019. Different letters above the columns indicate significant differences (*P* < 0.05) determined by a Duncan’s test between treatments. The same letters above the error bars indicate no significant difference at *P* < 0.05.

The initial fluorescence value (*F*_o_) increased with the aggravation of drought stress (Fig. 4a,b). In 2018, the *F*_o_ under LS, MS and SS was 9.03%, 9.89% and 14.13% higher than that of the CK, respectively (*P* < 0.05) (Fig. 4a). In contrast, the *F*_o_ only increased significantly under MS and SS, and the values were 24.84% and 50.92% higher than those of the CK in 2019, respectively (*P* < 0.05) (Fig. 4b). The *F*_o_ was higher in all the treatments in 2019 than in 2018 (Fig. 4a,b). The *F*_m_ decreased with the increase in drought stress (Fig. 4c,d). The difference of *F*_m_ under the LS and CK was not significant in 2018, while the *F*_m_ under MS and SS was 8.49% and 19.73% lower than that of the CK, respectively (*P* < 0.05) (Fig. 4c). In 2019, the *F*_m_ under LS, MS and SS were 10.02%, 15.83% and 21.89% lower than that of the CK (*P* < *0.05*) (Fig. 4d). The *F*_m_ was higher in 2018 under all the treatments than that in 2019 (Fig. 4c,d). The variable fluorescence of PS II (*F*_v_) decreased significantly under drought stress (Fig. 4e,f). The *F*_v_ values under LS, MS and SS were 19.05%, 37.62% and 92.86% lower than those in the CK in 2018, respectively (*P* < 0.05), and the values were 43.68%, 49.43% and 72.41%, respectively, in 2019 (Fig. 4e,f). The *F*_v_ in each treatment was higher in 2018 than in 2019 (Fig. 4e,f). Drought stress significantly decreased the activity of PS II (Fig. 4g,h). The *F*_v_/*F*_o_ values under LS, MS and SS were 16.24%, 25.41% and 46.82% lower than that in the CK in 2018, respectively (*P* < 0.05), and the values were 27.24%, 47.44% and 66.99% in 2019, respectively (Fig. 4g,h). The *F*_v_/*F*_o_ was lower in 2019 for each treatment than in 2018 (Fig. 4g,h). The *F*_v_/*F*_m_ values under drought stress were lower than that of the CK (Fig. 4i,j). There was no significant difference between the LS and CK (*P* > 0.05), while the values under MS and SS differed significantly compared with that of the CK (*P* < 0.05), which decreased by 10.37% and 24.12%, respectively (Fig. 4i). In 2019, the *F*_v_/*F*_m_ under LS, MS and SS decreased by 13.3%, 22.44% and 36.83%, respectively (Fig. 4j). The *F*_v_/*F*_m_ was lower in 2019 for each treatment than that in 2018 (Fig. 4i,j).

### Effects of drought stress on the main economic characters

With the aggravation of drought stress, the plant height of naked oats gradually decreased (Table 2). There was no significant difference between the LS and CK for plant height during the two-year period, while the height decreased by 6.30% and 8.01% in 2018 under MS and SS, respectively (*P* < 0.05), and the height decreased by 7.88% and 22.16% in 2019, respectively (*P* < 0.05) (Table 2). The plant height was almost the same under the CK in two years, while the height was higher under LS and MS in 2018 than in 2019, and it was higher under SS in 2019 (Table 2). Drought stress significantly decreased the panicle length (Table 2). Under LS, the panicle length decreased slightly for the two years, while the length decreased by 16.37% and 12.52% under MS and SS in 2018, respectively (*P* < 0.05), and decreased by 17.29% and 12.28% in 2019, respectively (*P* < 0.05) (Table 2). The panicle length was longer under all the treatments in 2018 compared with that in 2019 (Table 2). The spikelet number under LS and MS did not differ significantly from the CK, while the number under SS decreased by 12.6% and 21.9% (*P* < 0.05) in 2018 and 2019, respectively (Table 2). There was more spikelet in 2019 under all the treatments than in 2018 (Table 2). The grain weight per panicle under LS, MS and SS were 21.86%, 31.25% and 34.38% lower, respectively, (*P* < 0.05) than the CK in 2018, while the grain weight per panicle under LS did not differ significantly from the CK in 2019. However, the grain weight per panicle decreased by 21.88 and 53.13% under MS and SS, respectively in 2019 (Table 2). For both seasons, the grain weight per panicle in 2019 was more than that in 2018 under the CK, LS and MS, and the grain weight per panicle was lower under SS (Table 2). There was no significance difference between the stem number under LS and CK in both years (*P* > 0.05), while the stem number under MS and SS decreased by 11.24% and 15.73%, 16.17% and 33.03% in 2018 and 2019, respectively (Table 2). The stem number in 2019 was less than that in 2018 under all the treatments (Table 2).

**Table 2:** Effects of drought stress on the economic characteristics of naked oats.

Year	Treatment	Plant height (cm)	Panicle length (cm)	Spikelet number	Grain weight per panicle (g)	Stem number
2018	CK	64.0 ± 2.6 a	13.9 ± 0.5 a	30.9 ± 0.3 a	3.2 ± 0.1 a	44.5 ± 1.7 a
	LS	62.5 ± 2.3 ab	14.7 ± 0.2 a	28.7 ± 2.1 a	2.5 ± 0.2 b	42.5 ± 0.9 ab
	MS	59.0 ± 1.2 b	11.6 ± 0.4 b	28.5 ± 1.4 a	2.2 ± 0.2 bc	39.5 ± 2.3 bc
	SS	49.8 ± 3.4 c	12.1 ± 1.0 b	24.2 ± 1.4 b	2.1 ± 0.1 c	37.5 ± 1.5 c
2019	CK	64.7 ± 5.0 a	13.3 ± 0.6 ab	32.6 ± 1.3 a	3.3 ± 0.1 a	43.3 ± 5.0 a
	LS	58.7 ± 2.3 ab	13.7 ± 1.5 a	31.3 ± 3.1 ab	3.1 ± 0.3 a	39.3 ± 2.5 ab
	MS	53.3 ± 2.9 b	11.0 ± 1.0 c	28.9 ± 0.9 ab	2.5 ± 0.1 b	36.3 ± 2.9 b
	SS	54.7 ± 5.0 b	11.7 ± 0.6 bc	28.5 ± 2.4 b	1.5 ± 0.3 c	29.0 ± 1.0 c

Different letters above the columns indicate significant differences (*P* < 0.05) determined by a Duncan’s test between treatments. The same letters above the error bars indicate that there was no significant difference at *P* > 0.05.

### Effects of drought stress on the yield components

There was no significant difference in the number of panicles per pot among the drought stress treatments in 2018, while only SS significantly decreased the panicle number by 28.7% compared with the CK in 2019 (Table 3). There were more panicle numbers per pot in 2019 than in 2018 (Table 3). The performance of grain number per panicle differed between the two years, and the grain number per panicle decreased by 12.55%, 24.32% and 34.21% under LS, MS and SS in 2018, respectively, while the grain number per panicle only decreased significantly under SS in 2019 (*P* < 0.05) with a value of 34.4% (Table 3). The number of grains per panicle was less under the CK and SS in 2019 than in 2018, while it was higher under LS and MS in 2019 (Table 3). The 1000-grain weight decreased significantly under MS and SS with a decrease of 14.5% and 16.8% in 2018, respectively, and the values were 12.2% and 18.4% in 2019, respectively (Table 3). The 1000-grain weight was higher in 2019 than that in 2018 under all the treatments (Table 3). The yield decreased by 12.7%, 26.9% and 44.1% under LS, MS and SS in 2018, respectively (Table 3). In 2019, the decrease in yield was not significant under LS, while the yield decreased by 16.85% and 47.76% under MS and SS, respectively (Table 3). The yield was higher in 2018 than in 2019 under the CK and SS, and it was higher in 2019 under LS and MS (Table 3). Drought stress significantly decreased the biomass in 2018 (*P* < 0.05), and the biomass decreased by 6.39%, 18.3% and 21.3% under LS, MS and SS, respectively (Table 3). In 2019, the decrease in amount of biomass was not significant under LS, and the biomass decreased by 14.75% and 17.69% under MS and SS, respectively (Table 3). The biomass was higher in 2019 than that in 2018 under all the treatments (Table 3). Drought stress also significantly decreased the harvest index (*P* < 0.05) in both years, and the harvest index was higher in 2018 than that in 2019 under the CK, LS and SS, and it was higher under MS in 2019 (Table 3). The naked oat TOL under LS, MS and SS was 14.4, 30.6 and 50.1 g/pot in 2018, while the TOL was lower under LS and MS in 2019 with values of 10.5 and 18.5 g/pot. The TOL was higher under SS in 2019 with a value of 52.5 g/pot (Table 3). The naked oat MP under LS, MS and SS in 2018 was 106.2, 98.1 and 88.4 g/pot, while the MP was lower in 2019 under LS and SS with values of 104.8 and 83.8, and the MP was higher in 2019 under MS with a value of 98.1 (Table 3).

**Table 3:** Effects of drought stress on yield component, yield and biomass of naked oats.

Year	Treatment	Panicle number per pot	Grains per panicle	1000-grain weight (g)	Yield (g/pot)	Biomass (g/pot)	Harvest index	Tolerance index (TOL) (g/pot)	Mean Productivity (MP) (g/pot)
2018	CK	26.0 ± 2.6 a	148.7 ± 6.2 a	22.0 ± 1.0 a	113.39 ± 4.97 a	323.50 ± 3.40 a	0.35 ± 0.02 a	–	–
	LS	27.3 ± 1.2 a	130.1 ± 12.7 b	20.1 ± 1.1 ab	99.04 ± 3.96 b	302.83 ± 8.34 b	0.33 ± 0.01 ab	14.4	106.2
	MS	29.3 ± 1.5 a	112.6 ± 5.3 c	18.8 ± 0.7 b	82.80 ± 5.87 c	264.23 ± 5.49 c	0.31 ± 0.03 b	30.6	98.1
	SS	26.7 ± 2.1 a	97.9 ± 2.7 d	18.3 ± 1.3 b	63.34 ± 2.29 d	254.58 ± 15.40 c	0.25 ± 0.01 c	50.1	88.4
2019	CK	38.3 ± 5.7 a	141.3 ± 10.0 a	22.8 ± 0.5 a	110.0 ± 7.4 a	327.2 ± 22.01 a	0.34 ± 0.00 a	–	–
	LS	35.7 ± 3.1 a	138.7 ± 9.9 a	21.5 ± 1.5 ab	99.5 ± 13.12 ab	306.3 ± 19.97 ab	0.32 ± 0.04 a	10.5	104.8
	MS	32.7 ± 3.1 ab	129.0 ± 6.1 a	20.0 ± 1.9 bc	91.5 ± 9.85 b	278.9 ± 14.89 b	0.33 ± 0.05 a	18.5	100.8
	SS	27.3 ± 3.1 b	92.7 ± 6.1 b	18.6 ± 1.9 c	57.5 ± 5.22 c	269.3 ± 34.87 b	0.21 ± 0.02 b	52.5	83.8

Different letters above the columns indicate significant differences (*P* < 0.05) determined by a Duncan’s test between treatments. The same letters above the error bars indicate that there was no significant difference at *P* > 0.05.

### Relationship between the yield and photosynthetic characteristics of naked oat

**Figure 5:**
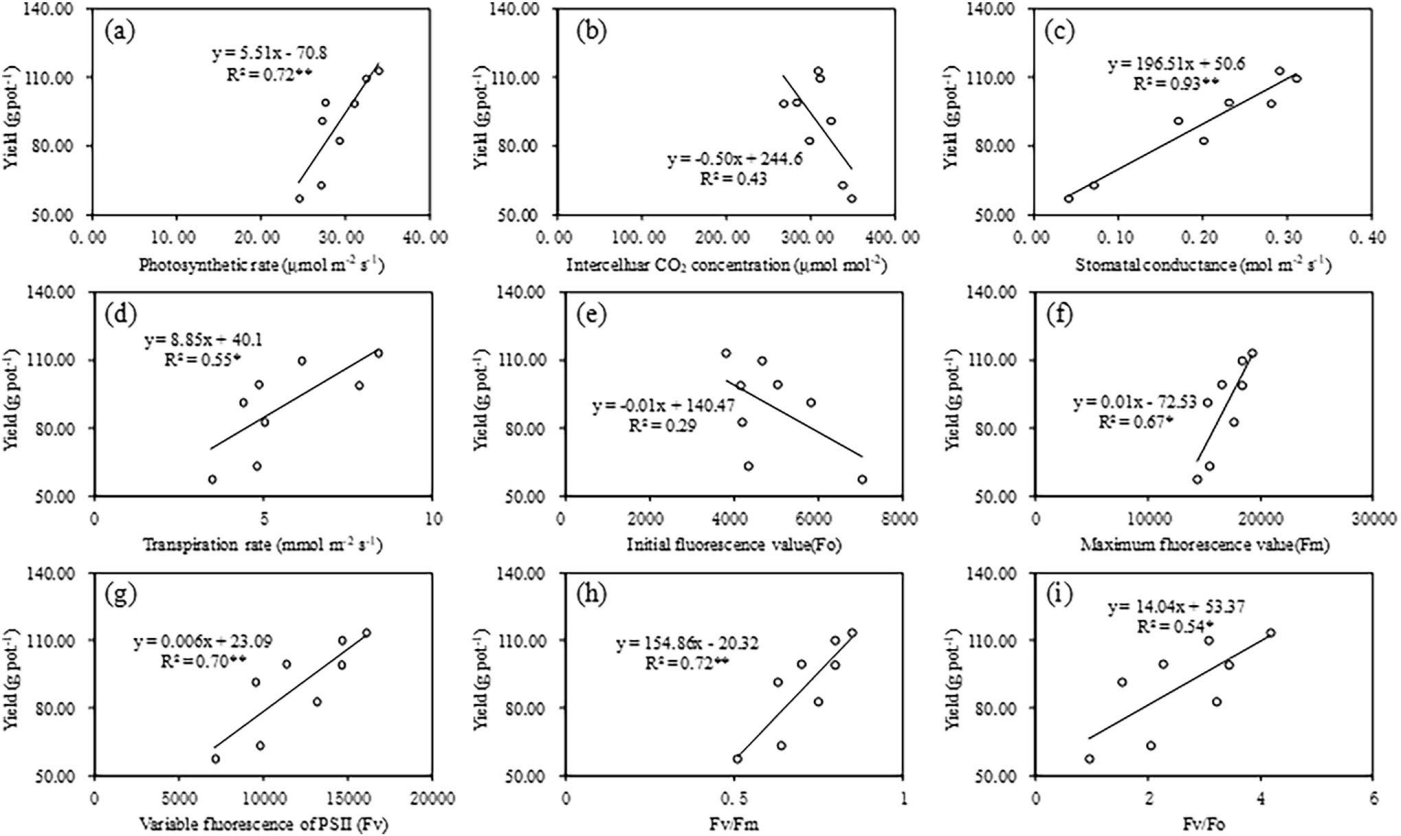
Relationship between the naked oat yield and photosynthetic rate (**a**), intercellular CO_2_ concentration (**b**), stomatal conductance (**c**), transpiration rate (**d**), initial fluorescence value (*F*o) (**e**), maximum fluorescence value (*F*m) (**f**), variable fluorescence of PSII (**g**), *F*v/*F*m (**h**) and *F*v/*F*o (**i**) in the Bashang area, Hebei Province, China. The solid line represents the linear trend for each variable. *P < 0.05. **P < 0.01.

The relationship between the yield of naked oat and its photosynthetic characteristics is shown in Fig. 5. The yield of naked oat increased significant with the increase in photosynthetic rate (*P* < 0.01), and the coefficient of determination (R^2^) between the rate of photosynthesis and yield of naked oat was 72% under drought stress (Fig. 5a). There was no significant relationship between the yield of naked oat and the intercellular CO_2_ concentration (Fig. 5b). Stomatal conductance and transpiration both had significant positive impacts on the yield of naked oat (*P* < 0.01) (Fig. 5c,d). There was no significant relationship between the yield of naked oat and *F*_o_ (*P* > 0.05) (Fig. 5e). The parameters of PS II, including *F*_m_ (*P* < 0.05), *F*_v_ (*P* < 0.01), *F*_v_/*F*_m_ (*P* < 0.01) and Fv/Fo (*P* < 0.05), all have significantly positively relationship with the yield of naked oat (Fig. 5f–h).

## Discussion

### The response of photosynthetic efficiency to drought stress

Photosynthesis is the basis for crop growth and development, and it is the major factor that determines the composition of crop productivity^24,25^. The decrease in photosynthetic rate under drought stress is a common phenomenon^26^. The *G*_s_ and *P*_n_ were found to decrease sharply with the increase in drought stress. The same phenomenon was found in this study because a decrease in the supply of water decreases the *G*_s_ under drought stress to reduce water loss, and stomatal closure further leads to an insufficient supply of CO_2_, thus, resulting in the reduction of *P*_n_^19,27,28^. The trends of change of *P*_n_, *G*_s_ and *C*_i_ enabled the determination of whether the stomatal factors are restricted^29^. During the early stage of drought stress (or light drought stress), the stomata closed first to reduce water transpiration, thus, preventing CO_2_ from entering the leaves^27^. Under moderate and severe drought stress, the concentration of *C*_i_ gradually increased as the *P*_n_ and *G*_s_ decreased, indicating that non-stomatal restriction may gradually became the primary factor of the decrease of photosynthetic rate as the drought stress deepens, which could be owing to the damage of chloroplast structure^30^. However, these conclusions merit further research, which could entail the use of ^13^C or ^18^O isotope tracers^31^.

### The impacts of drought stress on photosystem II

Chlorophyll fluorescence can reflect the primary photosynthetic reaction process, including the absorption of light energy and the transmission of excitation energy and photochemical reaction^32^. The degree of damage caused by stress can be reflected by measuring chlorophyll fluorescence parameters, such as the light utilization in PS II^33^. Under drought stress, plants maintain the balance of water budget by reducing the *T*_r_, which is an adaptive way to avoid drought^34^. The *F*_o_ reflects the degree of damage to the thylakoid membrane with more serious damage of the thylakoid membrane, which induces higher *F*_o_ values^34^. The *F*_m_ reflects the electron transfer through PS II. A lower *F*_m_ reflects a higher degree of thermal damage^34^. *F*_v_/*F*_o_ represents the potential activity of PS II and reflects the activity of PS II center^28^. The *F*_v_/*F*_m_ of plants is typically 0.75–0.85 under non-stress conditions^35,36^, and it will be significantly reduced under conditions of adversity or injury^37^. Our results of the two-year study showed that the *F*_o_ in oat leaves increased in parallel with the water stress during the critical period because the damage of degree of thylakoid in leaves increased gradually as the water stress deepened. The electrical transmission through PSII in the leaves was inhibited under drought stress conditions, and thus, the *F*_m_ decreased under water stress. Moreover, water stress reduced the efficiency of capturing light energy in the PSII reaction center of leaves. Thus, the *F*_v_/*F*_o_ and *F*_v_/*F*_m_ in the oat leaves were lower than those in the CK. In addition, more parameters, such as non-photochemical quenching (NPQ) and the fast repetition rate (F_m-FRR_), were related to PSII^38,39^. Under drought stress conditions, the intensity of fluorescence decreased, while the relative fluorescence of L-and K-bands in *Plectranthus scutellarioides* increased^40^. The same phenomenon was also found in other crops^41,42^. The regulation of drought stress for PSII could not be revealed comprehensively with the parameters measured in this study^43^. Nevertheless, this study could explain gaps in the knowledge of the impacts of drought stress on naked oat photosynthesis and yield formation.

Different cultivars typically responded differently to drought stress. Previous studies showed that the *P*_n_ of Bayou3 and Mengyan1 decreased by 42.7% and 34.2% under SS^5,6^, and their level of decline was more dramatic than that of Huazao 2 (used in this study). The *F*o increased by 39.0% and 20.3% for Bayou 3 and Mengyan 1, respectively, and the increase was higher than that for Huazao 2^5,6^. The changes of the parameters showed that the cultivar used in this study (Huazao 2) was more drought resistant than Bayou 3 and Mengyan 1.

### Effect of drought stress on economic and yield characters of naked oats

Drought stress has been shown to decrease wheat (*Triticum aestivum* L.) yield and composition factors^26,44^. In addition, the development of organs is significantly affected under drought stress^45^. In this study, the plant height, panicle length, panicle grain weight, stem number and 1000 grain weight of naked oats all gradually decreased under different amounts of drought stress. The same results were found in previous studies^30,46^. This is because drought stress decreases the accumulation of biomass, and thus, inhibits the growth of oat ears, leaves, stems and roots. However, the accumulation and distribution of dry matter were not determined in this study. Additional research should focus on the accumulation and distribution of dry matter of oat under drought stress conditions. Mild drought had little effect on oat biomass, while moderate and severe drought had a greater effect on oat biomass. Compared with light stress, the yield and yield components of oat decreased more under moderate and severe drought stress because the photosynthetic capacity of the leaves decrease more under moderate and severe drought stress.

The tolerance index (TOL) and mean productivity (MP) values provide information on the stability of yields under different conditions^22^. In this study, we analyzed the TOL and MP under different drought stress conditions in 2018 and 2019. The TOL was higher under LS and MS in 2018 than in 2019. The yield of naked oat was almost the same as that of the CK for both seasons. The higher TOL under 2018 was owing to the distribution of precipitation under LS and MS during the oat growing period. The TOL under SS was almost the same for both seasons, implying that the SS caused irreversible damage to the naked oat. The MP was higher in 2018 than in 2019 under LS and SS, and there was more precipitation during the growing season in 2018. This implies that the cultivar used was not very resistant to drought.

We further analyzed the relationships between the yield of naked oat and the photosynthetic characteristics, which showed that the photosynthetic rate was significantly related to oat yield (*P* < 0.01). Typically, *P*_n_ is an instantaneous value, and a higher *P*_n_ during the crop growth period might not produce higher yields ^47^. When the crops were subjected to stress, the activity of *P*_n_ was lower during the entire growing period, and the yield decreased significantly ^24^. The parameters related to PS II also significantly positively correlated with the yield of naked oat. In all, the drought stress decreases the efficiency of photosynthesis and the components of naked oat yield.

### Limitations of the study

There are some limitations in this study. First, the research was conducted on only one cultivar, and the amount of drought resistance varies for different cultivars. Therefore, more cultivars should be considered in future research. Secondly, previous studies showed that the indices, such as the activities of superoxide dismutase (SOD) and peroxidase (POD) and the content of malondialdehyde (MDA), would also change under drought stress conditions^48^. However, these parameters were not measured in this study. Future research should include more parameters. Owing to the limited conditions, the experiment only controlled the soil water content and established the degree of soil drought stress based on it. However, the actual degree of stress of the oat plants was not measured. In addition, only photosynthesis, fluorescence and laboratory tests were performed in this experiment, and changes in the physiological indices related to drought stress of oat were not measured. The next step is to determine the degree of stress of oat based on the leaf water potential and to determine the indicators related to drought stress related, such as the activities of superoxide dismutase (SOD) and peroxidase (POD) and the content of malondialdehyde (MDA) among others.

## Conclusions

Drought stress decreased the *P*_n_, *T*_r_, *G*_s_ under all the drought stress conditions during the critical period in which water was required. The *C*_i_ decreased under light LS, while it increased under MS and SS. The *F*_o_ and *F*_v_/*F*_m_ increased under drought stress, while the *F*_m_ decreased under drought stress. The oat yield decreased by 9.5–47.7% under drought stress conditions. Grains per panicle decreased 1.7–34.2% under drought stress conditions. The 1000-grain weight decreased by 5.7–19.1% under drought stress conditions. Drought stress had no significant impacts on the panicle numbers per pot. The *P*_n_, *T*_r_, and *G*_s_ all positively correlated with the yield of naked oat. The parameters of PSII, except for *F*_o_, all significantly positively correlated with the yield of naked oat (*P* < 0.05). This study provides additional knowledge on the different levels of drought tress on the production of naked oats in the Bashang area.
